# Methods for dynamic synchrotron X-ray respiratory imaging in live animals

**DOI:** 10.1107/S1600577519014863

**Published:** 2020-01-01

**Authors:** Kaye Susannah Morgan, David Parsons, Patricia Cmielewski, Alexandra McCarron, Regine Gradl, Nigel Farrow, Karen Siu, Akihisa Takeuchi, Yoshio Suzuki, Kentaro Uesugi, Masayuki Uesugi, Naoto Yagi, Chris Hall, Mitzi Klein, Anton Maksimenko, Andrew Stevenson, Daniel Hausermann, Martin Dierolf, Franz Pfeiffer, Martin Donnelley

**Affiliations:** a School of Physics and Astronomy, Monash University, Wellington Road, Clayton, VIC 3800, Australia; b Institute for Advanced Study, Technische Universität München, Garching Germany; c Chair of Biomedical Physics and Munich School of BioEngineering, Technische Universität München, 85748 Garching, Germany; d Robinson Research Institute, University of Adelaide, SA 5006, Australia; e Adelaide Medical School, University of Adelaide, SA 5000, Australia; f Respiratory and Sleep Medicine, Women’s and Children’s Hospital, 72 King William Road, North Adelaide, SA 5006, Australia; g SPring-8, Japan Synchrotron Radiation Institute, Kouto, Hyogo, Japan; h Imaging and Medical Beamline, The Australian Synchrotron – ANSTO, 800 Blackburn Road, Clayton, VIC 3168, Australia

**Keywords:** inverse Compton source, phase-contrast imaging, mouse, rat, trachea, lungs, airways, phase contrast, biomedical imaging

## Abstract

Small-animal physiology studies are typically complicated, but the level of complexity is greatly increased when performing live-animal X-ray imaging studies at synchrotron and compact light sources. This group has extensive experience in these types of studies at the SPring-8 and Australian synchrotrons, as well as the Munich Compact Light Source. These experimental settings produce unique challenges. Experiments are always performed in an isolated radiation enclosure not specifically designed for live-animal imaging. This requires equipment adapted to physiological monitoring and test-substance delivery, as well as shuttering to reduce the radiation dose. Experiment designs must also take into account the fixed location, size and orientation of the X-ray beam. This article describes the techniques developed to overcome the challenges involved in respiratory X-ray imaging of live animals at synchrotrons, now enabling increasingly sophisticated imaging protocols.

## Abstract 2

The imaging of live animals at a synchrotron source presents challenges in terms of remote monitoring and intervention, in addition to a sample that is changing and moving with time. This work describes experimental techniques that have been developed to address these challenges and capture image sequences that can equip a range of biomedical studies.

## 1. Background   

X-ray imaging is widely used to non-invasively reveal internal body structures, and can provide better spatial resolution than other non-invasive methods. These capabilities have seen X-ray imaging become an essential diagnostic tool in medical clinics, and support innovative research studies that examine both living and non-living samples.

Conventional X-ray imaging utilizes the attenuation of incident X-rays to create a ‘shadow’ image, where strongly attenuating features such as bones are revealed. In the last two decades, new methods of phase-contrast X-ray imaging (PCXI) have been developed, which can also reveal those weakly attenuating features that make up the rest of the body. These PCXI methods are particularly sensitive to interfaces between soft tissue and air, which means that the lungs and airways can be clearly visualized.

One challenge of imaging living soft tissue structures like the lungs is the continual motion of the tissue, requiring that images are captured rapidly to avoid a blurry image. This challenge is further compounded in small animals such as mice, often used in research studies, since a detector with reduced pixel size will require longer exposures to achieve the same statistics in each pixel and hence the same image quality. This is due to both a decreased area in which to collect photon statistics and, in the case of an optically coupled detector system, the requirement for a thin scintillator to avoid resolvable scintillator-associated blurring. Small pixels also mean that a visually imperceptible movement of only a few micrometres can result in motion blur across a number of pixels. These difficulties are addressed by high-flux synchrotron X-ray sources, which are capable of providing very high intensity, small-area X-ray beams compared with conventional X-ray sources (Suzuki *et al.*, 2004 ▸). This high flux enables researchers to capture high-speed movies to visualize not just the structure but also the function, by capturing movements or changes in the tissue that either are, or can be, a proxy for tissue and organ function. A second advantage of synchrotron X-ray beams is the associated high coherence. High spatial coherence (a relatively uniform phase across the wavefront) and, to some degree, high temporal coherence (a narrow range of X-ray wavelengths) result in improved imaging sensitivity. This means that smaller and more subtle features can be seen in the sample.

There are several advantages to X-ray imaging, both in the conventional attenuation mode and with phase contrast. Unlike nuclear methods, X-ray imaging does not require a radionuclide, although X-ray contrast agents can be used to highlight the cardiovascular system. X-ray imaging can typically provide better spatial resolution [below 10 µm (Lovric *et al.*, 2016 ▸)] than nuclear (PET/SPECT) or magnetic resonance imaging (MRI) [each limited to around 1 mm (Lizal *et al.*, 2018 ▸; Uecker *et al.*, 2010 ▸)] and typically with a shorter exposure time (Conway, 2012 ▸; Dournes *et al.*, 2016 ▸; Lizal *et al.*, 2018 ▸), making it possible to capture small and fast-moving structures. The key disadvantage to X-ray imaging is the associated ionizing radiation dose, which is normally monitored and minimized. This is a particular consideration with high-flux synchrotron small-animal imaging, where a large dose can mean that researchers, and/or institutional and facility ethics boards, may require that animals are humanely killed after imaging. However, experiments can be designed such that the radiation doses are kept to acceptable levels to enable longitudinal studies to be performed (Donnelley, Morgan, Siu, Fouras *et al.*, 2014 ▸).

Since air/tissue interfaces provide such strong contrast with synchrotron PCXI, and because our work has focused on respiratory tract imaging, this manuscript will focus on imaging of the lung parenchyma and conducting airways. However, many of the live-animal techniques we describe here are translatable to other organs.

Synchrotron X-ray imaging of small animals can be used to advance research studies in a number of ways, and we mention just a few of the many here. The first category of respiratory studies are those aiming to better understand physiology or disease. Examples of research to date include examining the first breaths after birth (te Pas *et al.*, 2009 ▸), the role of the heart in mixing air in the lungs (Dubsky *et al.*, 2018 ▸), the inflation of individual alveoli (Lovric *et al.*, 2017 ▸) and understanding normal mucociliary clearance of particles (Donnelley *et al.*, 2012 ▸). The second category of studies are those aiming to test the effects of medical treatment or intervention. This has included visualizing the delivery of treatments (Donnelley *et al.*, 2013 ▸; Porra *et al.*, 2018 ▸; Gradl *et al.*, 2019*a* ▸) and the effect of treatments on a micrometre-scale [*e.g.* on mucociliary clearance (Donnelley, Morgan, Siu, Farrow *et al.*, 2014 ▸) or airway surface liquid depth (Morgan *et al.*, 2014 ▸; Luan *et al.*, 2017 ▸)] and the effect of interventions at a whole-organ scale [*e.g.* high-frequency ventilation (Thurgood *et al.*, 2012 ▸) or methacholine challenge (Bayat *et al.*, 2009 ▸)]. Although some physiological processes repeat and hence present multiple opportunities for imaging [*e.g.* the breath cycle (Gradl *et al.*, 2019*b* ▸)], often a treatment-induced change is not easily repeated, and requires fast and reliable imaging without any opportunity to repeat an exposure. The third category of studies looks at the diagnostic power of synchrotron imaging, often with an aim to then translate the technique to a more compact X-ray source that can be easily integrated into hospitals and clinics, particularly for lung imaging (Fouras *et al.*, 2012 ▸; Gromann *et al.*, 2017 ▸). For a complete review of synchrotron-based lung imaging studies, see Bayat *et al.* (2018 ▸). Note that there are some diagnostic synchrotron X-ray imaging applications that progressed directly to human diagnostics, without first establishing methodologies in small animals, such as mammography (Stampanoni *et al.*, 2011 ▸; Castelli *et al.*, 2011 ▸).

In 2010 we described the challenges of performing live-animal imaging experiments at synchrotrons (Donnelley *et al.*, 2010 ▸) and detailed a range of techniques designed to increase experiment success. Here we provide an update that draws on our subsequent experience to describe new methodologies and our suggestions for optimal imaging protocols. These protocols have been developed and tested in mice, rats and pigs, but could also be applied to other species depending on the capabilities of the specific facility. The techniques described have been developed at the SPring-8 synchrotron in Japan, at the Imaging and Medical Beamline (IMBL) at the Australian Synchrotron, and at the Munich Compact Light Source (MuCLS) in Germany.

## 2. Animal considerations   

All experimental protocols using animals must typically be approved by the researcher’s ‘home’ animal ethics committee, as well as that of the imaging facility. Experiments must always be designed according to the principles of animal welfare and the three Rs (reduction, refinement and replacement), and must ensure that experimental animals do not suffer discomfort, distress or pain, and that the animals remain sufficiently anaesthetized throughout the experiments. This is particularly important when using paralytic agents such as pancuronium bromide – for example, to prevent muscle activity or spontaneous respiration – since their use makes it more difficult to determine if an animal is sufficiently anaesthetized.

### 2.1. Animal monitoring   

**Figure 1:**
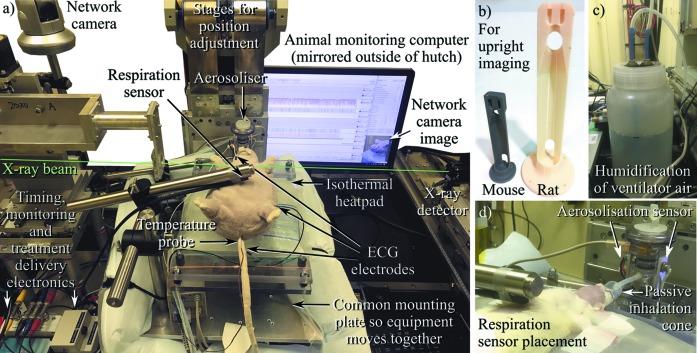
Experimental setup for remote high-magnification X-ray imaging of small animals in a prone position, shown here at BL20XU of the SPring-8 synchrotron. (*a*) Here, the animal (a soft toy in this image) is set up for free-breathing imaging, with ECG, respiration, temperature and appearance monitored and displayed using PowerLab, both inside and outside the experimental hutch. (*b*) For upright imaging, a mouse or rat is placed in these mounts, with incisors placed on a loop of thread (not shown) and supporting tape placed around the abdomen. There is the possibility to clip the ventilation hub into the mount, particularly useful for tomographic imaging. (*c*) Air into a ventilator is humidified using a bubbler system. (*d*) During free-breathing imaging, aerosolized treatment can be inhaled via a cone attached to the nebuliser. An LED/photodiode-based sensor detects when aerosol is passing out of the nebuliser. Note also the placement of the respiration chest motion sensor.

**Figure 2:**
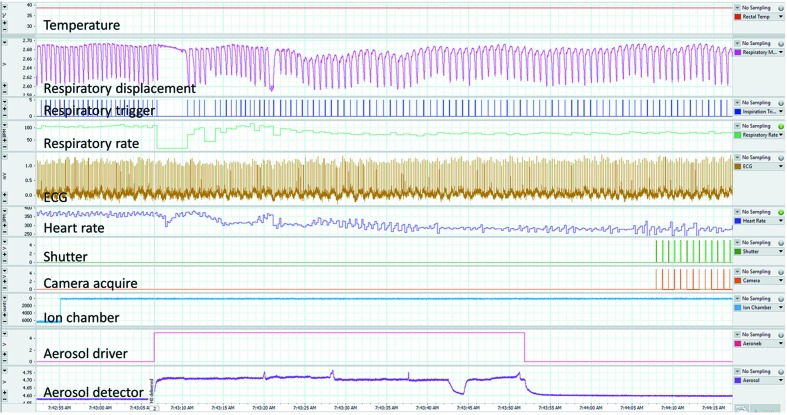
An example of the physiological monitoring performed using the *LabChart* system from ADInstruments. For this experiment the chest motion respiratory monitor was used (pink channel 3) to provide a trigger signal (blue channel 4) that drives the shutter (green channel 8) and camera (orange channel 9) to acquire images at the same point in every breath. The aerosol detector (purple channel 11) confirms that treatment is delivered when the aerosol control signal (pink/red channel 10) is on. Note that (*a*) the ion chamber is placed before the fast shutter in this case, but can also be placed after the fast shutter to directly measure the dose to the animal; (*b*) it is not unusual for the animal to hold its breath briefly at the start of aerosol delivery.

Synchrotron facilities are not typically designed specifically for animal experiments, so customized animal health monitoring systems adapted to the local facility and beamline environments are essential. Since the animals are isolated in a radiation-shielding enclosure during imaging, high-magnification remote-controlled IP cameras (*e.g.* Panasonic BB-HCM580) can be used to visually confirm the animal’s status throughout the experiments (see Fig. 1 ▸). A range of experimental parameters can also be monitored and recorded using a data acquisition system such as the PowerLab hardware and *LabChart* software (ADInstruments), combined with appropriate sensors and amplifiers (*e.g.* ECG BioAmp). These allow the animal’s temperature (rectal probe), oxygen saturation, respiration waveform, respiratory rate and ECG to be measured, with an increase in heart rate used as one indicator of reducing anaesthesia depth. Experimental parameters including shutter status (opened/closed), image acquisition, ion chamber output and treatment delivery can also be recorded. An example *LabChart* data recording is shown in Fig. 2 ▸. This system allows comprehensive remote monitoring of the animal and image acquisition parameters from outside the X-ray imaging radiation enclosure.

### 2.2. Anaesthesia   

The choice of anaesthetic for imaging studies is important, and our recommendations have changed since our previous publication (Donnelley *et al.*, 2010 ▸). Although it is possible to use inhalable anaesthetics such as isoflurane to provide deep and stable anaesthesia for long periods, this can add a substantial extra layer of complexity to the experimental protocols. This is because the isoflurane supply must be carefully maintained at the animal preparation stage, during transfer into the X-ray imaging radiation enclosure, and throughout the experiment. The use of long-acting injectable anaesthetics reduces these logistical requirements, but the choice of injectable anaesthetic will often be determined by what is available and/or common practice in the experiment location. In Australia a common anaesthetic for rodents is a mix of medetomidine and ketamine (Jang *et al.*, 2009 ▸), but the need for a narcotic handling licence complicates ketamine use in Japan. There we have used pentobarbital sodium (Nembutal/Somnopental) for anaesthesia induction and maintenance, via a continuous intraperitoneal delivery using a syringe pump, but this drug has a short duration of action and small therapeutic index, too frequently resulting in either light anaesthesia or overdose. More recently, a combination of medetomidine, midazolam and butorphanol has proven suitable for rats and mice (Kawai *et al.*, 2011 ▸; Kirihara *et al.*, 2016 ▸). The reversal agent atipamezole can also be used for recovery experiments using either of the medetomidine-based anaesthetic mixes. Our experience suggests that, provided adequate depth of anaesthesia is maintained, for many study designs it is not necessary to paralyse animals (*e.g.* with pancuronium bromide) to acquire high-quality images of the functioning respiratory system.

### 2.3. Ventilation   

For respiratory imaging studies, a key question is whether animals must be artificially ventilated. If the study demands that every breath must be identical, that a particular rate or pressure are used, that breath-holds are included, or if respiratory mechanics need to be measured, then a ventilator is essential. Our previous manuscript described the use of the flexiVent system (Scireq, an Emka Technologies company) for rodent imaging studies. Although this system is considered the gold standard for lung-function assessment (essential when establishing new imaging measures of lung function), we experienced some difficulties in using it to perform complex imaging protocols that involve irregularly timed image capture. An alternative is the Accuvent 200 (Notting Hill Devices, Melbourne, Australia), which is specifically designed for X-ray imaging studies by synchrotron-experienced scientists and engineers. This unit allows pressure-controlled ventilation at a range of respiratory rates and pressures, with a positive inspiratory pressure of 14 cm H_2_O and positive end-expiratory pressure of 2 cm H_2_O commonly used in rodents. Ventilators designed for human use can be used for large animals such as pigs or sheep. Either tracheostomy or orotracheal intubation can be used for airway access, but intubation (using 20 Ga or 16 Ga intravenous cannulas as endotracheal tubes in mice and rats, respectively) is rapid, minimally invasive and repeatable (Cmielewski *et al.*, 2017 ▸), although it may not provide the tight seal required for assessing lung mechanics in circumstances where a full range of airway pressures are required. A short (<1 mm) sleeve of tight-fitting silicone tubing stretched over the tip of the ET cannula can be used to improve the seal when placed just above or just below the vocal chords during intubation. However, if artificial ventilation is not essential, but each breath must be accurately identified for image acquisition, then respiration can instead be detected in rodents using a non-contact fibre optic displacement sensor (RC-60, Philtec, MD) (Burk *et al.*, 2012 ▸), or in large animals by using respiratory bands (AD Instruments). We connect the displacement sensor to a passive RC bandpass filter (0.75–25 Hz) to minimize noise and prevent the signal drifting over time if the animal moves away from the sensor. An image of the displacement sensor used for detecting chest respiration movements in a rat is shown in Fig. 1 ▸(*d*).

### 2.4. Body temperature   

Maintaining a physiological environment throughout the preparation and imaging procedures is vital for accurate measurements. While anaesthetized, the body temperature of the animal can drop rapidly, so maintaining temperature is important at every step after anaesthesia induction begins. For small animals this may be achieved using an infrared heat lamp (noting the light can interfere with the non-contact respiratory sensor unless well shielded), or, depending on the animal orientation during imaging, more stable control can be obtained by lying the animal on a Deltaphase Isothermal Pad (Braintree Scientific, USA), which maintains a constant normal body temperature for up to 4 h. For longer imaging studies we have designed a system to hold rats and mice in a supine position on an isothermal pad on the imaging stage (see Fig. 1 ▸). The addition of a forced-air patient warming system such as the Bair Hugger (3M, unit model 77510) can also assist the maintenance of normal body temperature. The possibility of resulting thermal effects on the precision stages should be considered, but we have generally found the bulk movement of the animal is the most significant effect on position stability.

### 2.5. Air humidification   

For respiratory studies it is also necessary to maintain adequate airway hydration while animals are intubated or tracheostomized. A ventilated animal experiences reduced inspired-air humidity because the intubation means that inspired air bypasses the nose, the primary organ responsible for airway humidification. Rats and mice naturally breathe through their noses rather than their mouths (Agrawal *et al.*, 2008 ▸), and thus tracheal air humidity is considered to be close to 100%, regardless of the environmental humidity. In the often-dry environments of synchrotron facilities, or when supplying dry gas to the ventilator, inspired air can be humidified using a simple water-bubbler on the ventilator input [see Fig. 1 ▸(*c*)]. Alternatively, the use of a respiratory detector for image capture triggering is also an effective way to maintain air humidity by avoiding artificial ventilation.

### 2.6. Radiation dose   

Synchrotron beamlines are typically designed to deliver high X-ray flux, hence while performing imaging experiments it is necessary to be mindful of the radiation dose, particularly for longitudinal recovery studies (Donnelley, Morgan, Siu, Fouras *et al.*, 2014 ▸). For high-magnification experiments in small animals (see Section 3 below) the use of a 6 mm fast X-ray shutter (*e.g.* Uniblitz XRS6 and VMM-T1 driver, Vincent Associates, NY, USA, which has an opening time of <5 ms) ensures that the animal is only irradiated while the camera is acquiring images, thus significantly reducing the total dose. Larger aperture shutters (*e.g.* Uniblitz XRS25) can be substituted when a larger field of view is required.

## 3. Imaging considerations   

**Figure 3:**
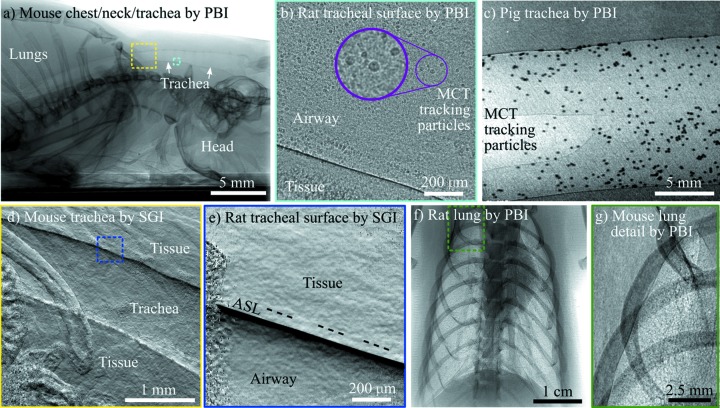
Typical *in vivo* X-ray phase-contrast images of the respiratory system. (*a*) Using propagation-based imaging (PBI), the full length of a mouse trachea from the lungs (left) to the mouth (right) is visible. Coloured boxes are indicative of the approximate imaging locations of the panels shown with corresponding colour. PBI reveals the airway surfaces and mucociliary transit (MCT) tracking particles at high resolution, captured here in (*b*) a rat and (*c*) a pig. Alternatively, single-grid imaging (SGI) can be applied to capture an image sensitive to the derivative of the phase, which reveals the curvature of the trachea in (*d*) a mouse (tiled image), and, at higher magnification (blue box), reveals (*e*) the airway surface liquid (ASL), shown here in a rat (note the ‘saturated’ phase signal near the ASL/Airway edge has been set to black). (*f*) PBI enhances the visibility of the lungs, providing (*g*) a sharp boundary around the edge and a speckle pattern from the air sacs in the lung, shown here in (*f*) a rat and (*g*) a mouse. In all cases shown here, the image capture was synchronized to the breath. Note that respiratory images captured in mice and rats are very similar, simply scaled for the animal size. Panel (*a*) was collected at 25 keV on BL20B2, and panels (*b*), (*d*) and (*e*) on BL20XU at the SPring-8 synchrotron. Panels (*c*) and (*f*) were captured at 55 keV on the IMBL at the Australian Synchrotron, and panel (*g*) was captured at 25 keV at the Munich Compact Light Source (cropped here from the full image to show detail).

Selection of the appropriate imaging facility and beamline is driven by a range of factors, but the most important two are the field of view that is required and the available flux density. The required field of view will be determined by the physiological process or organ that is to be imaged, and may vary from the cellular level to the whole animal. Synchrotron X-ray sources have a low divergence, so the beam size does not change significantly through the imaging setup. This means that the beam size defines the accessible field of view, and, unlike a laboratory X-ray source, there is limited scope for accessing a smaller/larger area of illumination by moving the sample closer to or further from the X-ray source unless other specialized optics for magnification or focusing are added. The widest beam is found at the Australian Synchrotron’s IMBL (∼30 cm across, 3 cm tall, 135 m from the source), which can be used to image large animals using a patient table attached to a robotic arm (Donnelley *et al.*, 2019 ▸), translating the animal and detector vertically through the beam if a taller field of view is required. The BMIT-BM beamline at the Canadian Light Source provides a beam that is only a little smaller. When imaging a mouse or rat lung, synchrotron beamline designs such as those employed at the IMBL (Murrie *et al.*, 2015 ▸; Stevenson *et al.*, 2017 ▸), SPring-8’s BL20B2 (Goto *et al.*, 2001 ▸), SLS’s Tomcat (Stampanoni *et al.*, 2006 ▸), ESRF’s ID17 (Thomlinson *et al.*, 2000 ▸) or the SSRF’s BL13W1 (Xie *et al.*, 2013 ▸) are ideal choices. An alternative to conventional synchrotron X-ray sources is a more compact source that collides the electron beam with a laser to produce X-rays via the inverse Compton effect. An early example of this technology is the Munich Compact Light Source (developed and manufactured by Lyncean Technologies Inc., USA), which includes a dedicated imaging beamline with two end-stations [designed and installed by researchers of the Technical University of Munich (Eggl *et al.*, 2016 ▸)], and provides illumination over an area suitable for small-animal lung imaging. For high-magnification X-ray microscopy, *e.g.* examining the surface of the airway, smaller-area beamlines such as SPring-8’s BL20XU (Suzuki *et al.*, 2004 ▸) are a more suitable choice. The flux density must be high enough to facilitate sufficiently short exposure times to avoid motion blur, and in some cases even more rapidly to capture dynamics. This flux density requirement means that the majority of the dynamic small-animal imaging work described here has been performed either at a synchrotron or a compact synchrotron (Gradl *et al.*, 2018 ▸; Loewen, 2004 ▸). Examples of the types of images acquired on several of the mentioned beamlines are shown in Fig. 3 ▸.

All synchrotron imaging beamlines are typically capable of producing monochromatic X-ray beams at energies that are suitable for imaging biological samples and producing phase contrast. Note, however, that some spread of energies will not significantly reduce the phase contrast quality (Wilkins *et al.*, 1996 ▸). Imaging of mice and rats is typically performed at 25–30 keV (Siu *et al.*, 2008 ▸; Suzuki *et al.*, 2002 ▸), with larger animals such as pigs requiring 35–60 keV (Wiebe *et al.*, 2015 ▸; Donnelley *et al.*, 2019 ▸). These energies balance sufficient penetration of the X-ray beam through the animal to the detector, maximized at high energies, with the phase effects and absorption contrast which are seen predominantly at low energies. The X-ray dose delivered to the animal should also be considered when choosing the X-ray energy and the number and duration of X-ray exposures planned for the study.

If the researcher would like to see soft tissue structures like the airways, and hence phase contrast is desired, then the spatial coherence of the X-ray source should also be considered [unless using a method of phase contrast like grating interferometry that can include a third grating to increase the effective spatial coherence (Pfeiffer *et al.*, 2006 ▸)]. Almost all synchrotron sources [including inverse Compton scattering driven compact synchrotrons (Eggl *et al.*, 2016 ▸)] produce highly coherent light as a result of a large source-to-sample distance (approximately tens to hundreds of metres) relative to that used at a conventional X-ray source (∼1 m), which is possible without significant flux loss due to a highly collimated synchrotron beam. Alternatively, a smaller source size than what is used in conventional sources (*e.g.* <10 µm) will provide high coherence, as seen with micro-focus sources (Larsson *et al.*, 2011 ▸).

Unlike a portable X-ray source that can be positioned in any orientation, a synchrotron X-ray beam has a fixed position and a horizontal (or close-to-horizontal) direction of propagation, which can affect the design of experiments. Careful positioning of the animal is therefore essential in producing a clear view of the relevant anatomy, and to minimize the presence of overlying bone, other organs and tissues in the beam. To acquire anterior–posterior (AP) images of the lungs of rodents, the animals must be held head high, suspended by their incisors, which we have achieved with the use of custom-designed 3D printed holders [Fig. 1 ▸(*b*)] to adequately support the weight of the animal, since this is not a normal physiological position for these animals. We have successfully used this method for mouse and rat studies at multiple facilities, including for the acquisition of computed tomography (CT) projection images. However, this orientation is likely to be logistically difficult for live large-animal imaging studies due to the weight of the animal, although it has been used for post-mortem CT acquisitions in pigs (Donnelley *et al.*, 2019 ▸; see Section 5 below). If one wishes to image the tracheal airway surfaces, then a supine lateral view helps to avoid the X-ray beam passing through the spine. This results in a clearer image of the airways because the lateral beam only passes through soft tissues and not bone. This positioning approach has been used to image the airway surface of mice, rats and pigs.

**Figure 4:**
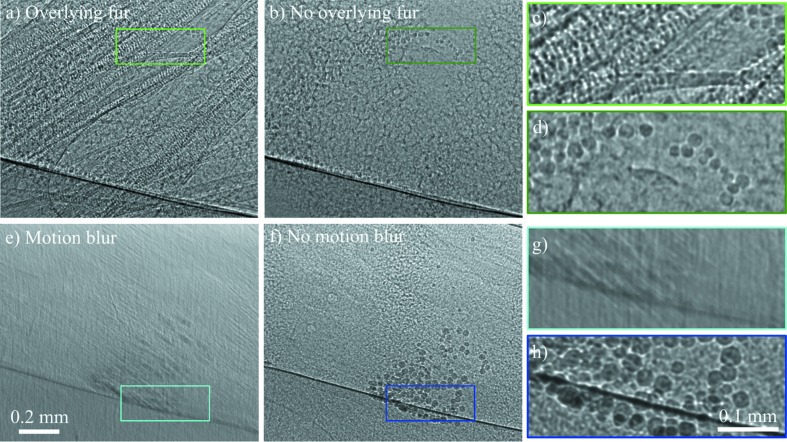
The surface of the trachea of a rat with instilled particles (for mucociliary clearance tracking), demonstrating the detrimental effects of motion blur and overlying structures such as fur when imaging at high resolution. Each artefact makes it impossible to resolve individual particles. (*a*) Stray fur from another part of the rat obstructs the field of view (where the fur has actually been removed), compared with (*b*) a clear image when the fur has been combed away from the field of view. 25 ms exposures. Panels (*c*) and (*d*) show magnified sections of (*a*) and (*b*), respectively. Note that some beads have moved during the 2 min taken to comb/smooth the fur away from the field of view. (*e*) Motion blur of around 20 µm during a 50 ms exposure compared with (*f*) a sharp image taken less than 2 s later, again with a 50 ms exposure, but with the image capture synchronized to the stationary part of the breath cycle. Panels (*g*) and (*h*) show magnified sections of (*e*) and (*f*) respectively. All images were taken with 25 keV synchrotron X-rays from BL20XU at the SPring-8 synchrotron.

For high-magnification studies in rodents, such as imaging the tracheal airway surface, there are additional challenges. Our studies at BL20XU at the SPring-8 synchrotron use a field of view of ∼1 mm × 1 mm with 0.5 µm pixels, so searching for a specific small anatomical region of interest within a large animal requires substantial operator expertise and knowledge of anatomical landmarks. This process can be slow and results in extra radiation dose to the animal during the search procedure. At these high levels of magnification the fur and skin can also cause strong phase contrast effects that obscure the features of interest. Our previous recommendation was that hairless strains of animals could be used (Donnelley *et al.*, 2010 ▸), but these animals exhibit other physiological differences, and relying on them precludes the use of other disease models (*e.g.* the cystic fibrosis mice and rats relevant to our research interests). However, the detrimental effects of overlying fur and skin can be minimized by removing fur from the imaging area using a combination of clippers (Neuro blade; CareFusion, San Diego, CA, USA) followed by a depilatory cream (Nair, Church & Dwight, Australia), and the addition of a thin layer of petroleum jelly to reduce the texture of the skin/air interface (Vaseline, Unilever, Australia). This method is rapid, effective and produces little adverse reaction. An example of the difference in image quality when fur is present is shown in Figs. 4 ▸(*a*)–4(*d*).

Although the trachea of mice, rats and pigs can be imaged in this manner, it remains difficult to visualize the surface of lower airways because of the strong signal from surrounding lung tissue and the constant motion from respiration and heartbeat. At high levels of magnification, small animal movements (*e.g.* 20 µm of movement with 0.5 µm pixels) produce motion artefacts that can render acquired images unusable. Respiratory artefacts can be minimized using a gating strategy to acquire images at the same point(s) within the breath cycle, typically in the end-expiratory phase where there is less motion. Respiratory gating can be performed prospectively or retrospectively, but retrospective gating dramatically increases the number of images (together with radiation dose) that must be acquired, and can potentially increase post-processing complexity as the images must be binned into the correct phase of respiration. Again, when imaging the whole lung, contrast-based measures of lung inflation can be used for retrospective gating, but this is far more challenging to perform at the tracheal scale. Prospective gating removes these challenges, with image acquisition driven by an inspiration signal from either a ventilator or respiration detector. The difference between gated and ungated images is shown in Figs. 4 ▸(*e*)–4(*h*).

**Figure 5:**
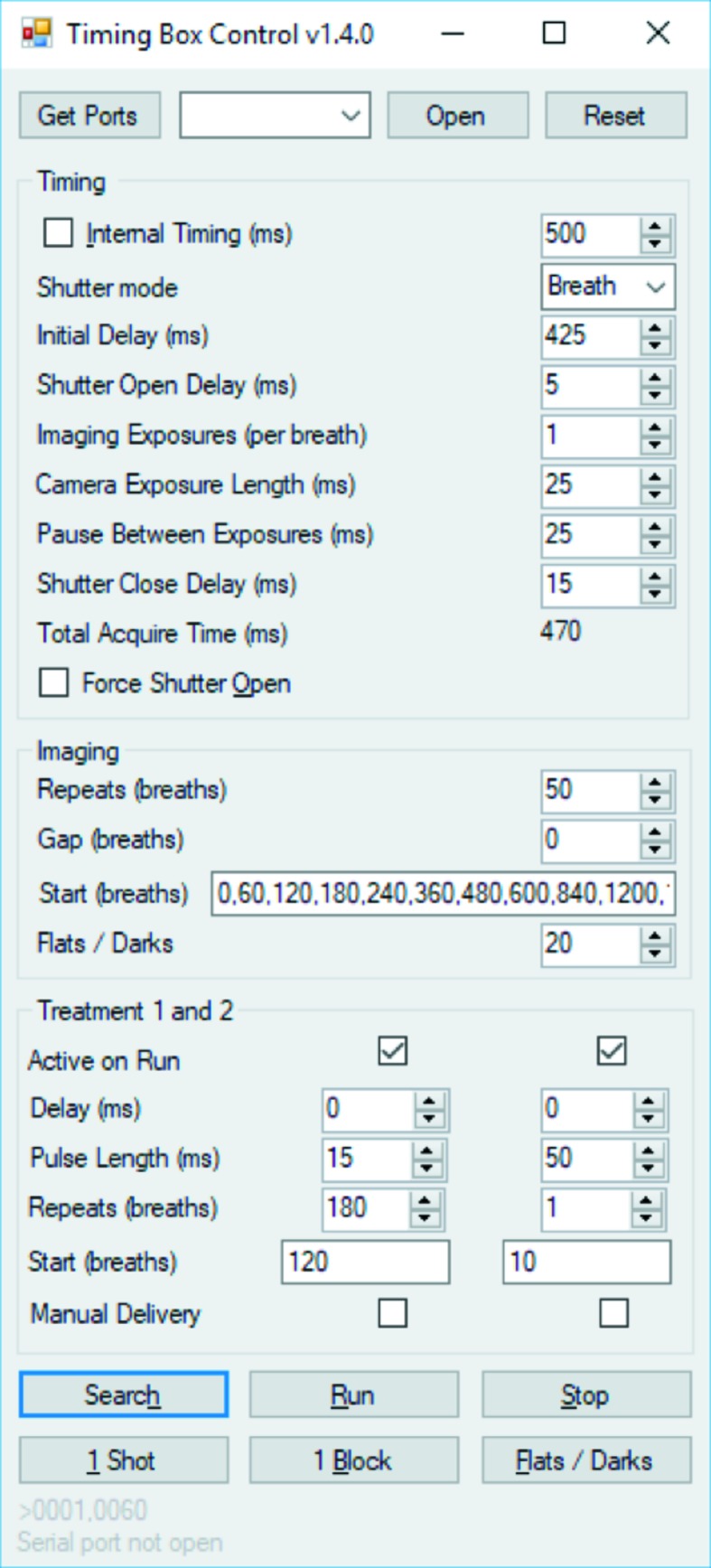
Timing box control screen. The firmware and software are freely available by contacting the authors.

Maintaining control of all aspects of experiment timing is essential. To achieve this we have created an Arduino-based ‘timing box’ and ‘control graphical user interface’ (Fig. 5 ▸) which make experiment timing accurate, reliable and simple. The unit can use internal timing or external timing signals from the ventilator or respiration detector. It controls all aspects of fast X-ray shutter and image acquisition timing, can acquire blocks of shuttered images at specified intervals, and can drive two outputs for the delivery of test substances (see Section 4 below) at required times. The inclusion of a button to acquire single exposures means that the anatomical region of interest can be located with the minimal number of images, and therefore the lowest possible dose.

In order to observe the airways and other soft tissue structures, we take advantage of the high coherence from synchrotron sources to create phase contrast. The most easily implemented form of phase contrast is propagation-based imaging (PBI), where a distance of several centimetres to metres is introduced between the animal and the detector. Anatomical features will introduce variations in the phase of the X-ray wavefield, and the waves will self-interfere during propagation, creating high-contrast dark/bright bands (Cloetens *et al.*, 1996 ▸). Because there is a significant difference in X-ray phase properties between air and tissue, the edges of the airways in particular will be enhanced as dark/bright bands. This modality is useful because the raw images are easy to read (visually similar to a conventional X-ray image, but with edge enhancement around soft tissue) and only one exposure is typically required for quantitative measurements [*e.g.* lung air volume (Kitchen *et al.*, 2008 ▸)]. The propagation distance from the animal to the detector should be chosen to balance contrast, maximized at larger distances, with spatial resolution, which suffers at larger distances [see Figure 50.4 in the work by Anastasio & Riviere (2012 ▸)]. In the following text we provide some explanation for the propagation distances chosen for various applications and animals, detailed in the papers cited below, and summarized in Table 1 ▸. Note that these distances depend on pixel size and X-ray energy, which we have also stated in Table 1 ▸. We have found that rat/mouse/rabbit pup lung imaging is best with a propagation distance of around 2 m at a synchrotron (Kitchen *et al.*, 2004 ▸), slightly less at a compact synchrotron where penumbral blurring is a consideration (Gradl *et al.*, 2018 ▸), and slightly more at a micro-focus source where penumbral blurring is small and magnification reduces the relative phase effects at a given distance (Preissner *et al.*, 2018 ▸). For high-resolution imaging of the airway surface, where a small pixel size captures narrow bright/dark bands, distances can be as short as 10 cm for a rat trachea and 30 cm for a mouse trachea (where the projected tissue thickness of the smaller airway will contain weaker gradients). If there are introduced features that do not produce such a strong signal, like inhaled beads or debris, or if the signal-to-noise ratio of the imaging setup is low, a slightly longer propagation distance may be preferred (Donnelley, Morgan, Siu, Fouras *et al.*, 2014 ▸). With a larger animal (*e.g.* a pig or sheep), a larger field of view is likely desired and hence larger pixels are used. A longer propagation distance is then required to see the same level of contrast, and hence we used 2.5 m when imaging clearance of inhaled beads in the airways of large animals (Donnelley *et al.*, 2017 ▸, 2019 ▸).

Although an increased propagation distance will improve the sensitivity to subtle changes in composition or thickness, there may be some cases where propagation-based phase contrast is not sufficiently sensitive to differentiate the targeted anatomy. One example of this is differentiating the liquid lining of the airways from surrounding tissue (Morgan, Paganin, Parsons *et al.*, 2012 ▸). In such a case, a form of phase contrast that is sensitive to the first derivative of the phase may be used, achieved using either an analyser crystal (Ingal & Beliaevskaya, 1995 ▸), a grating interferometer (Momose *et al.*, 2003 ▸; Weitkamp *et al.*, 2005 ▸), an edge-illumination setup (Olivo & Speller, 2007 ▸) or a single-grid (Bennett *et al.*, 2010 ▸; Morgan *et al.*, 2010 ▸) or speckle-tracking (Bérujon *et al.*, 2012 ▸; Morgan, Paganin & Siu, 2012 ▸) setup. These setups can also provide a ‘dark-field’ signal that originates from sub-pixel structures (Arfelli *et al.*, 2000 ▸; Chapman *et al.*, 1997 ▸; Pfeiffer *et al.*, 2009 ▸) and which is not accessible using propagation-based PCXI. Many of these phase derivative/dark-field approaches require several exposures of the sample, each with a slightly different optical setup, in order to reconstruct an image. This is difficult if the sample is moving and hence appears differently in each of the exposures. In the case where the sample motion is not repeated [*e.g.* a response to treatment (Morgan *et al.*, 2014 ▸)], it is possible to use a single-exposure single-grid imaging (SGI) setup, which captures all the required information in one exposure, at the cost of spatial resolution. In the case where sample motion is cyclic [*e.g.* the breath cycle (Gradl *et al.*, 2019*b* ▸)], we have captured the additional exposures required for grating interferometry in subsequent cycles.

A key consideration related to the number of exposures is the length of the exposures, particularly to avoid motion blur [Fig. 4 ▸(*e*)]. Once anaesthetized, mice naturally breathe at 90–150 breaths min^−1^, rats at ∼80 breaths min^−1^ and pigs at ∼15 breaths min^−1^. When imaging a free-breathing or ventilated mouse (choosing 120 breaths min^−1^) breath-to-breath at SPring-8 BL20XU, we typically use an exposure time of around 25 ms, adjusting the delay from the start of the breath (‘Initial delay’ in Fig. 5 ▸) so that the image is captured during the most stationary part of the breath cycle. Using a ventilator, it is possible to add a ‘breath hold’ that keeps the airways still for longer, or to ventilate at a reduced breath rate during image capture (Gradl *et al.*, 2018 ▸), useful at sources that produce less flux. Note that there may be a further limitation introduced if there is motion that is on a shorter scale than the breath, like inhaled beads clearing quickly along the airway or the heart beating close to the area of interest. If the imaging seeks to capture the breath itself, then the exposure times should be short enough to capture around 15 images per breath, *e.g.* 10 ms (Murrie *et al.*, 2015 ▸). In particular, fast imaging may also be limited by the time taken for the fast shutter to open and close or the time for the camera to write the images. In such cases, it can be better to leave the shutter open (‘Shutter mode’ in Fig. 5 ▸) or use a camera with onboard memory to store images that are then saved later.

## 4. Research study considerations   

The particular study design will introduce a range of considerations, including how any test substances (*e.g.* pharmaceuticals) will be delivered, and how long and how often imaging should occur. For respiratory studies, three modes of substance delivery are normally used; aerosolization, liquid bolus delivery and dry powder delivery:

(i) Aerosols can be generated using a range of devices, but the Aeroneb (Aerogen, Galway, Ireland) vibrating mesh devices are particularly useful due to their ability to efficiently aerosolize small quantities of fluid, and because their output is scalable for small animal studies, even though they are designed for human use. They can be integrated into the inspiratory line of a ventilator circuit, or a small flow of gas can be used to direct the aerosol over an animal’s nose using a nose cone for passive inspiration [Fig. 1 ▸(*d*)]. One weakness of these devices that we have noted is that at the gas flow rates used for rodents (significantly lower than for humans) the aerosolized fluid can condense into an obstructive droplet on the underside of the mesh, dramatically reducing aerosol output. Ordinarily, any condensation could be easily and directly monitored in benchtop studies, but in a synchrotron radiation enclosure this is difficult to achieve. To verify aerosol output through a Scireq Aeroneb adaptor we have created a simple optical measurement circuit consisting of an infrared light-emitting diode and photodiode placed on opposite sides of the aerosol delivery tube [Fig. 1 ▸(*d*)]. The output voltage of this circuit varies depending on the amount of aerosol passing through the tube, and this can be monitored and recorded in *LabChart* to verify correct aerosol delivery (Fig. 2 ▸).

(ii) A bolus delivery of treatment may be delivered to a number of sites: into the tissue/bloodstream, into the nasal airways or into the lungs. Intraperitoneal or subcutaneous liquid bolus drug delivery can be performed using an indwelling needle combined with a remotely actuated syringe pump system (*e.g.* UMP2, World Precision Instruments, Sarasota, FL, USA). When testing the effects of interventions such as pharmaceutical treatments, it is often necessary to be able to achieve agent delivery remotely, for example, after a short baseline imaging period.

Nasal airway fluid delivery can be performed using the same system attached to a fine polyethyl­ene cannula (*e.g.* PE10 for mice and rats) that is positioned using a micro-positioner so that the tip is sitting just within a nostril.

**Figure 6:**
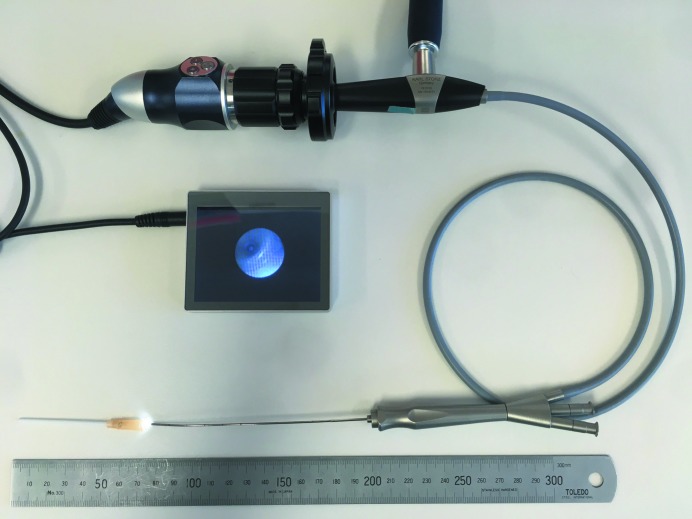
Rat bronchoscopy system. The system consists of a sialendoscope, USB endoscope camera, USB LED light and screen. The junction of the Luer hub and the 14 Ga intravenous cannula, used as an endotracheal tube, can be seen on the screen. An example video is available at https://www.youtube.com/watch?v=qCuW4k3D74U.

Fluid delivery directly to the lungs requires intubation of the animal. If imaging does not need to capture the moment of bolus delivery, the delivery can be efficiently performed before the animal is transferred to the imaging radiation enclosure, using a miniature bronchoscope system (McIntyre *et al.*, 2018 ▸) based on a clinical sialendoscope (Karl Storz 11582A, Tuttlingen, Germany). This bronchoscopy system has since been miniaturized using a portable, low-cost, high-definition USB endoscopy camera, 3.5 inch screen, and high-intensity USB LED light source (MicroTech Medical, Hangzhou, China) that enables video capture during procedures (see Fig. 6 ▸). Fluids, or other substances such as agar beads (Growcott *et al.*, 2011 ▸), can be delivered directly into the trachea or to at least the fourth generation of the lungs in rats using the bronchoscope fluid channel. If the lung bolus delivery must occur during imaging, then the animal should be intubated, with a small diameter heat-thinned polyethyl­ene cannula inserted through a small hole in the silicone tubing just above the endotracheal tube (above the mouth) and run non-obstructively through that tube to the lung or trachea (Donnelley *et al.*, 2010 ▸). The delivery can then be remotely actuated using the syringe pump system mentioned earlier in this section.

(iii) Dry powder delivery. For some studies it is necessary to deliver dry powders to the airways. This can be performed using a Dry Powder Insufflator (DP-4 with AP-1 air pump, previously available from PennCentury, Wyndmoor, PA, USA); but in our experience, effective and reliable lung delivery requires a high level of operator expertise and regular practice. The very small quantities delivered are highly variable, the stainless steel tip moves during delivery using the supplied hand pipettor, and those devices are no longer available for purchase. A more robust and predictable solution involves loading the particles into the tip of a miniature bronchoscope (McIntyre *et al.*, 2018 ▸), which is connected to an air supply (*e.g.* 400 kPa) via a pneumatic valve (Clippard EVO-3-12-L-M15, Cincinnati, OH, USA). The particles can be loaded by dipping the tip 1–2 mm into the powder, then wiping the excess particles from the outside of the tip, verifying the quantity remaining using an optical microscope. Powder delivery is performed by opening the air valve electronically via the timing hub described above (*e.g.* 25 ms pulse), allowing delivery during imaging. This method has been validated in rat airways, but has also been tested using a long delivery cannula (SurgiVet Bronco Alveolar Catheter, CBAL5190, Smiths Medical, MN, USA) placed into a pig trachea (Donnelley *et al.*, 2019 ▸).

The imaging stage setup is important. During high-resolution imaging studies it is recommended that all relevant support equipment (*e.g.* Aeroneb with nose cone, chest motion sensor, animal monitoring leads) are attached to the *XY* stage so that they move with the animal. On BL20XU this has been achieved by adding a large mounting plate to the stage to hold all the support equipment and the animal [see Fig. 1 ▸(*a*)]. For CT acquisitions the ventilator tubing must be carefully managed during stage rotation to maintain a good connection to the animal and prevent it from altering the position of the animal. This can be achieved using a mounting design that secures the ET tube to the animal holder (4Dx Pty Ltd), combined with long ventilator tubing run vertically to a secure point high above the centre of the rotation stage. This setup provides the tubing enough space to twist without impeding rotation or reducing airflow.

## 5. Post-mortem imaging   

Although dynamic live-animal imaging is essential for understanding many physiological processes, the relevant anatomy can sometimes be better visualized post-mortem when there is no movement present. We have performed high-resolution CT imaging (HRCT) in intact animals shortly after they have been humanely killed, but the results can be variable due to slow slumping of the animal carcass caused by muscle relaxation and gravity, and potentially *rigour mortis* if extended imaging times are required. This slow motion of the organs during projection acquisition causes blurring in the reconstructed images. Rather than imaging the whole animal when only a specific organ is of interest, it can be better to remove the organ of interest, immediately embed it in low-temperature-setting agarose, and then acquire images as soon as the agar has set. In the case of the lungs, the buoyancy of the air-filled lungs provides an extra challenge. To perform HRCT of rodent lungs we inflate the lungs to a desired pressure (*e.g.* 10 cmH_2_O), tie off the trachea with a long length of suture to maintain this pressure, excise the lung, feed the suture into a small hole in the bottom of a specimen container and seal the hole with tape, and fill the container with warm 2% agarose. The lung then floats upside-down in the agarose until it solidifies. These lung samples are typically ready for HRCT imaging approximately 20 min after death. Once imaging is complete, the organs can be easily broken out from the agar and fixed for histological analysis. An alternative method involves drying the excised tissue as described by Harbison & Brain (1983 ▸).

## 6. Discussion   

PCXI is a powerful technique for studying live-animal anatomy and physiology, and particularly for respiratory imaging, since there are few other non-invasive imaging modalities that can provide this sensitivity, speed and resolution. Dynamic live-animal imaging at high-flux synchrotron facilities is clearly challenging, but studies can be successful when using appropriate techniques. The greatest improvements in capability we have introduced since our first description of live-animal imaging techniques (Donnelley *et al.*, 2010 ▸) are: the development of easily configurable hardware capable of performing accurate timing; the use of more physiologically accurate imaging conditions (*e.g.* the removal of artificial ventilation for airway surface imaging studies, and improved temperature and humidity maintenance); better remote animal monitoring capabilities; and more stable anaesthesia regimens. With these improvements a range of complex experiments to examine the structure and function of the respiratory system are now possible.

## Figures and Tables

**Table 1:** Parameters for propagation-based phase-contrast X-ray imaging of small animals The field of view was typically 2000 pixels across. The pixel size used for imaging the trachea depends on whether the images aim to capture the whole width of the airway (*e.g.* to observe mucociliary clearance) or a single airway surface (*e.g.* to measure airway surface liquid depth).

Animal	Breathing rate (breaths min^−1^)	Imaging region	Exposure time (ms)	Energy (keV)	Propagation distance (m)	Pixel size (um)
Mouse	90–200	Nose	100	25	∼0.4	∼0.5
		Trachea	∼25	25	∼0.4	∼0.18–0.5
		Lungs	∼10 (<30)†	25	∼2–4	∼10
Rat	∼80	Trachea	∼50	25	∼0.4	∼0.36–1
		Lungs	∼20 (<50)	25	∼3–4	∼13–20
Pig	∼15	Trachea	∼100	55	∼4	∼7.6

† When using X-ray sources that have less flux than a synchrotron, a ventilator can be configured to briefly slow the respiratory rate so that exposure times for lung imaging can be extended up to 200 ms (Gradl *et al.*, 2018 ▸).
